# What it means to be alive: a synthetic cell perspective

**DOI:** 10.1098/rsfs.2023.0036

**Published:** 2023-08-11

**Authors:** Yuval Elani, John M. Seddon

**Affiliations:** ^1^ Department of Chemical Engineering, Imperial College London, Exhibition Road, London SW7 2AZ, UK; ^2^ fabriCELL, Imperial College London, Exhibition Road, London SW7 2AZ, UK; ^3^ Department of Chemistry, Imperial College London, 82 Wood Lane, London, W12 0BZ, UK

**Keywords:** biotechnology, synthetic cells, engineering biology

## Abstract

Advances in bottom-up synthetic biology offer the exciting—albeit contentious—prospect of transitioning bio-science researchers from passive observers of life to potential creators of it. Synthetic cells closely emulate the attributes of their biological counterparts. These rationally designed microsystems exhibit emergent properties and life-like functionalities. They can therefore be used as simplified cell models to decipher the rules of life, and as programmable biologically powered micromachines for application in healthcare and biotechnology more broadly. While there is a consensus that current synthetic cells are not yet ‘living’, the question of what defines ‘aliveness’ is gaining increasing relevance. Exploring this concept necessitates a multidisciplinary approach, where scientists from across domains in the physical, life, engineering and social sciences participate in community-level discussions, together with the acceptance of a set of criteria which defines a living system. Achieving a widely accepted definition of ‘living’ represents a possible mission-oriented endpoint to the synthetic cell endeavour, uniting the community towards a common goal. As the field evolves, researchers must address regulatory, ethical, societal and public perception implications, while fostering collaborative efforts to harness the transformative potential of synthetic cells.

## 1. Synthetic cells: a brave new ‘bio’ world

Advances in bottom-up synthetic biology have ushered in a new era of bio-design, transforming researchers from mere observers of life to potential creators of it. Synthetic cells, also known as artificial cells or protocells, are cell-like entities that closely resemble their biological counterparts in terms of building blocks, behaviours and morphological attributes. These cells are typically constructed using biomolecular components such as DNA, RNA, proteins, small molecules and lipids, but can also incorporate abiotic components and supramolecular machineries [1]. The assembly of these components into rationally designed microsystems leads to the emergence of new properties and life-like functionalities [2].

Since its nascent days in the late 1990s, the field of synthetic cells has experienced rapid development. In the past five years in particular, an influx of new tools, technologies, techniques and concepts from various branches of science and engineering has accelerated progress in this area. Researchers from diverse disciplines are now actively contributing to the field, and multinational consortia have been established to drive further advancements [3–5]. Recognizing its potential, research funders, academic institutions, industrialists and policymakers are increasingly acknowledging that creating synthetic cells from inanimate molecular building blocks—making life from non-living matter—represents one of the greatest scientific challenges of our time. At the forefront of this multidisciplinary endeavour lies Engineering Biology, a field that applies engineering principles to the design, construction, and modification of biological systems.

## 2. Why build synthetic cells?

Beyond pure scientific intrigue, the pursuit of building a synthetic version of a living cell is driven by an understanding-by-building philosophy, aimed at unravelling the fundamental rules governing life. Synthetic cells serve as invaluable tools for advancing our comprehension of biological processes, as they allow us to deconstruct cells into their modular components and explore the intricate webs of interactions that govern living systems [6]. Another significant driver, steadily gaining prominence, lies in the potential of synthetic cells as a new class of programmable biologically powered machines. These cells hold promise in various biotechnological applications, particularly in healthcare, sensing and environmental remediation [7,8].

## 3. What is living?

Away from the sciences however there are several philosophical implications of this research area. Primary among them is the age-old philosophical question: what does it mean to be alive? This question has been applied to varied research areas, including artificial intelligence, robotics, virtual worlds and simulations, all of which involve the creation of complex, dynamic systems with the potential to exhibit lifelike behaviours. When applied to the context of synthetic cells, the exploration of ‘aliveness’ takes on a unique character and was subject to intense debate during the discussions held at the Royal Society in London in November 2022. A clear consensus on the matter remains elusive, with various individuals proposing different definitions at different times. Broadly speaking, two main flavours of arguments have emerged from the discourse.

### 3.1. Autonomous existence

A cell is considered living when it exhibits autonomous self-replication, without the continuous need for human intervention, while sustainably acquiring and metabolizing components from its environment. It must possess the ability to adapt to varying surroundings and not be confined by the constraints of human-designed programming. Once a cell reaches the capability of self-replication and regeneration of its machinery, it can be likened to a ‘universal constructor’, akin to von Neumann's Self-Reproducing Automata scheme [9]. In this state, the cell becomes a machine capable of generating copies of itself [10], exemplifying the essence of life in its self-sustaining and evolving nature.

### 3.2. Biomimicry

A cell is deemed alive when it exhibits behaviours characteristic of living systems. Examples include metabolism, energy generation, motility, communication, division and evolution. In line with the central dogma of biology, the cell's capacity to pass on hereditary (genetic) information to its offspring over successive generations is also considered relevant. In relation to this viewpoint, ‘communication’ has been proposed as a potentially quantifiable measure of life-likeness [11]. A cell may be regarded as alive when biological cells cannot distinguish synthetic cells from naturally occurring ones, akin to a cellular-level Turing test [12]. The current research trend in the community follows a biomimetic approach, where academic groups strive to replicate behaviours associated with life in the context of synthetic cells, one behaviour at a time. Researchers are increasingly combining multiple life-like behaviours to create synthetic cells with enhanced complexity and functionality.

## 4. Is it living and why does it matter?

The question of ‘what is living’ holds not only philosophical significance but also has wider scientific implications. Firstly, if synthetic cells are used as models to approximate living cells, the closer they resemble their living counterparts, including their emergent properties, the more confidently we can rely on these models and draw conclusions from experimental findings.

Secondly, the definition of ‘living’ has regulatory implications. Current regulations governing engineered biological systems and genetically modified organisms (GMOs) apply to living entities. As synthetic cells are inanimate materials that do not self-replicate and lack vital signs of life, they do not fall under this category (at least for now). This quality is one of the attractions of using engineered synthetic cells in real-world applications. Related to this point is one of public perception and public acceptance of synthetic cell technology. Understandably, the highest level of public scepticism and societal resistance arises when the entity being engineered is alive, and therefore self-sustaining, autonamous, and evolving. Therefore, researchers in this field have a responsibility to proceed with caution, particularly given the rapid pace of advancements in this area.

Thirdly, achieving the milestone of creating something widely accepted as living can be seen as a natural endpoint in the quest to construct synthetic cells. This achievement has the potential to unite the research community and provide a clear goal, guiding them in a cohesive and well-coordinated manner. An affirmative answer to the question ‘is it living’ could serve as an answer to the broader question of ‘when are we done?’, as discussed Prof. Stephen Mann's commentary in this volume [13].

## 5. Summary

Although there is wide consensus that the synthetic cells currently under development in academic laboratories around the world are not yet living, we are entering an era where this question is becoming increasingly relevant. As such, a community-level discussion and acceptance of the criteria of life, and quantitative methods to assess the extent to which a system is indeed living, is needed. To do this, we will have to bring ethicists, philosophers and social scientists into the fold, and a concerted and community-wide effort needs to be established.

## Data Availability

This article has no additional data.

## References

[1] Buddingh BC, van Hest JC. 2017 Artificial cells: synthetic compartments with life-like functionality and adaptivity. Acc. Chem. Res. **50**, 769-777. (10.1021/acs.accounts.6b00512) 28094501 PMC5397886

[2] Guindani C, da Silva LC, Cao S, Ivanov T, Landfester K. 2022 Synthetic cells: From simple bio-inspired modules to sophisticated integrated systems. Angewandte Chemie **134**, e202110855. (10.1002/ange.202110855) PMC9314110 34856047

[3] Frischmon C, Sorenson C, Winikoff M, Adamala KP. 2021 Build-a-cell: engineering a synthetic cell community. Life **11**, 1176. (10.3390/life11111176) 34833052 PMC8618533

[4] Staufer O et al. 2021 Building a community to engineer synthetic cells and organelles from the bottom-up. Elife **10**, e73556. (10.7554/eLife.73556) 34927583 PMC8716100

[5] Schwille P et al. 2018 MaxSynBio: avenues towards creating cells from the bottom up. Angew. Chem. Int. Ed. **57**, 13 382-13 392. (10.1002/anie.201802288) 29749673

[6] Gaut NJ, Adamala KP. 2021 Reconstituting natural cell elements in synthetic cells. Adv. Biol. **5**, 2000188. (10.1002/adbi.202000188) 33729692

[7] Sato W, Zajkowski T, Moser F, Adamala KP. 2022 Synthetic cells in biomedical applications. Wiley Interdiscip. Rev.: Nanomed. Nanobiotechnology **14**, e1761. (10.1002/wnan.1761) PMC8918002 34725945

[8] Boyd MA, Kamat NP. 2021 Designing artificial cells towards a new generation of biosensors. Trends Biotechnol. **39**, 927-939. (10.1016/j.tibtech.2020.12.002) 33388162

[9] Neumann JV. 1966 Theory of self-reproducing automata. IEEE Trans. Neural Netw. **5**, 3-14.

[10] Lavickova B, Laohakunakorn N, Maerkl SJ. 2020 A partially self-regenerating synthetic cell. Nat. Commun. **11**, 6340. (10.1038/s41467-020-20180-6) 33311509 PMC7733450

[11] Lentini R et al. 2017 Two-way chemical communication between artificial and natural cells. ACS Cent. Sci. **3**, 117-123. (10.1021/acscentsci.6b00330) 28280778 PMC5324081

[12] Damiano L, Stano P. 2020 On the ‘life-likeness’ of synthetic cells. Front. Bioeng. Biotechnol. **8**, 953. (10.3389/fbioe.2020.00953) 32984270 PMC7479812

[13] Mann S. 2023 Cell mimicry: bottom-up engineering of life. Interface Focus **13**, 20230034. (10.1098/rsfs.2023.0034) PMC10415737 37577003

